# The Molecular Basis of Human Anophthalmia and Microphthalmia

**DOI:** 10.3390/jdb7030016

**Published:** 2019-08-14

**Authors:** Philippa Harding, Mariya Moosajee

**Affiliations:** 1 UCL Institute of Ophthalmology, London, EC1V 9EL, UK; 2 Moorfields Eye Hospital NHS Foundation Trust, London, EC1V 2PD, UK; 3 Great Ormond Street Hospital for Children NHS Foundation Trust, London, WC1N 3JH, UK

**Keywords:** anophthalmia, microphthalmia, coloboma, eye, genetics, development, induced pluripotent stem cells, SOX2, OTX2, genes

## Abstract

Human eye development is coordinated through an extensive network of genetic signalling pathways. Disruption of key regulatory genes in the early stages of eye development can result in aborted eye formation, resulting in an absent eye (anophthalmia) or a small underdeveloped eye (microphthalmia) phenotype. Anophthalmia and microphthalmia (AM) are part of the same clinical spectrum and have high genetic heterogeneity, with >90 identified associated genes. By understanding the roles of these genes in development, including their temporal expression, the phenotypic variation associated with AM can be better understood, improving diagnosis and management. This review describes the genetic and structural basis of eye development, focusing on the function of key genes known to be associated with AM. In addition, we highlight some promising avenues of research involving multiomic approaches and disease modelling with induced pluripotent stem cell (iPSC) technology, which will aid in developing novel therapies.

## 1. Introduction

**Figure 1:**
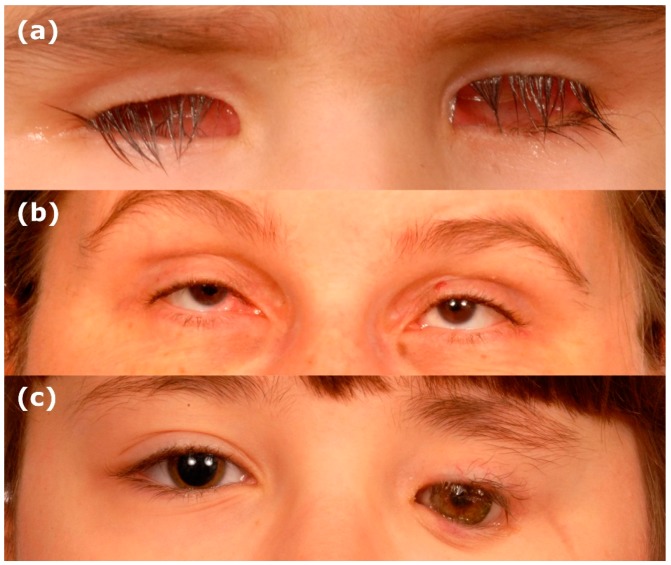
Clinical images of anophthalmia and microphthalmia. (**a**) Bilateral anophthalmia. (**b**) Bilateral microphthalmia. (**c**) Unilateral anophthalmia with shell.

The development of the human eye is a tightly controlled morphogenetic process which requires precise spatial and temporal gene regulation [1,2,3,4,5]. Perturbation of early eye organogenesis, either due to genetic or environmental factors, can result in lack of eye induction or abortion of eye development [6]. Anophthalmia and microphthalmia (AM) are, respectively, the absence or reduced size of the ocular globe compared to the population age-adjusted mean (Figure 1) [7,8]. Both are part of the same clinical spectrum and, therefore, can overlap, as the phenotype can be difficult to delineate in severe cases [9,10]. True anophthalmia occurs when eye development is aborted at the stage of the developing optic vesicle around 3–4 weeks gestation, leading to absence of the eye, optic nerve and chiasm, which can be confirmed through MRI brain and orbit scans [9]. Frequently, a small cystic remnant is detectable termed clinical anophthalmia, which occurs when the optic vesicle has formed but subsequently degenerates, hence a hypoplastic optic nerve, chiasm or tract may be present [10]. In microphthalmia, the eye has a smaller volume, and can be associated with a reduced corneal diameter (microcornea defined as a horizontal diameter <9 mm in a newborn and <10 mm in children >2 years) [11]. An axial length of <19 mm at 1 year of age or <21 mm in an adult using ultrasound B-scan substantiates a diagnosis of microphthalmia, representing ≥2 standard deviations below normal [11]. The estimated prevalence of microphthalmia and anophthalmia is 1 in 7000 and 1 in 30,000 live births, respectively [12]. The effect on vision is dependent on the severity, size of the eye and associated ocular malformations [9,10,11]. AM are considered part of the phenotypic continuum with ocular coloboma, a structural malformation resulting from incomplete fusion of the optic fissure, and are likely to have the same genetic basis [12,13,14,15]. Microphthalmia is reported in 3.2%–11.2% of blind children, but despite this there are no treatment options to improve visual function in microphthalmic patients, with current management focusing on maximising existing vision and enhancing cosmetic appearance [9,11,16].

AM has high clinical heterogeneity, and is rarely an isolated condition, often associated with other ocular abnormalities within the microphthalmic or contralateral eye (complex), such as anterior segment dysgenesis, ocular coloboma, cataract or vitreoretinal dysplasia (Table 1) [11]. Furthermore, 33%–95% of AM occurs alongside additional non-ocular systemic malformations, with 20%–45% of individuals diagnosed with a recognised syndrome [6,7,10,17,18,19]. The phenotypic variation observed with AM may be due to the multitude of genetic variants, including genetic modifiers as significant intrafamilial variability is seen, and/or environmental factors, such as maternal vitamin A deficiency or alcohol consumption [7,20,21,22,23]. The genetic variation is attributable to chromosomal and monogenic causes, with over 90 identified associated genes (Table 1). Genetic diagnosis can be obtained through molecular testing, such as array comparative genomic hybridisation (aCGH) or whole exome/genome sequencing [11]. Identifying the underlying genetic cause can influence appropriate disease management with recruitment of the correct multidisciplinary team to screen for potential syndromic associations, as well as guide genetic counselling and family planning [6,11]. The molecular diagnosis can be established in approximately 70% of bilateral anophthalmia or severe microphthalmia cases, but less than 10% of unilateral cases, which consists of the majority of patients [6,9,11,12]. Through understanding the genetic regulation underlying human eye development, and clarifying the roles of signalling molecules, we can elucidate how aberrant gene expression results in eye developmental disorders. Additionally, it is possible to better understand genotype–phenotype relationships, prognosis and develop new treatments.

## 2. The Genetic Basis of Human Eye Development

### 2.1. Eye Field Specification

**Figure 2:**
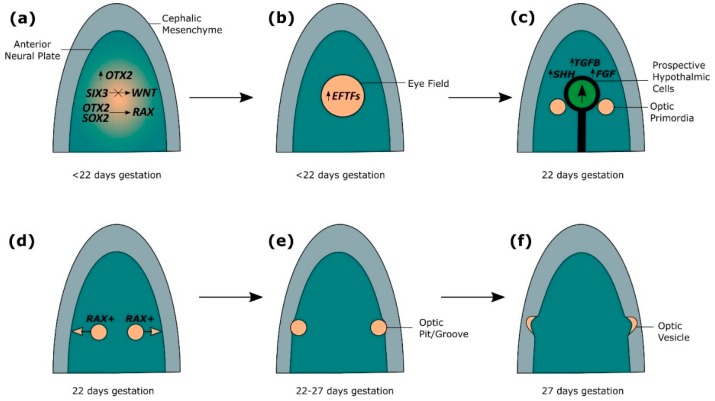
The genetics of early optic vesicle development. (**a**) A single eye field is induced at the midline of the anterior neural plate, through *OTX2* and *SOX2* coregulation of *RAX*, along with *SIX3*-mediated repression of *WNT*, which allows upregulation of eye field transcription factors (EFTFs) including *PAX6*, *RAX*, *LHX2*, *TBX3* and *SIX6*. (**b**) The eye field is formed expressing EFTFs, which form a self-regulating network sufficient to coordinate the development of the eye through suppression of genes that antagonize eye development and upregulation of genes required for eye development. (**c**) Secretion of factors from TGFβ, FGF and SHH families from the underlying axial mesoderm stimulate the anterior migration of prospective hypothalamic cells, causing the eye field to split in two to form bilateral optic primordia. (**d**) Cellular proliferation and bilateral migration of the optic primordia is regulated by *RAX*. (**e**) The optic primordia evaginate from the forebrain through the cephalic mesenchyme forming bilateral optic pits/grooves. (**f**) Extended evagination of the optic pits through the cephalic mesenchyme results in the formation of bilateral optic vesicles.

Human eye development is initiated at three weeks gestation in the anterior neural plate (Figure 2a) [2,24]. *OTX2* is required for the specification of the forebrain, and allows the neural plate to become competent for eye development by coordinating expression of eye field initiators *SIX3*, *RAX* and *PAX6* [2,3,24,25,26,27]. The role of *Otx2* in initiating eye development has been shown through animal studies investigating eye development, which have shown *Six3* activates *Pax6* and *Rx2* expression in medaka fish, and is sufficient to induce ectopic eyes in the presence of *Otx2* in medaka fish and *Xenopus* [2,3,24,25,26,27]. *OTX2* and *SOX2* coregulation of *RAX*, along with *SIX3*-mediated repression of *WNT* allows upregulation of eye field transcription factors (EFTFs), including *PAX6*, *RAX*, *LHX2*, *TBX3* and *SIX6* (Figure 2a,b) [1,2,25,28,29]. *Rax* has been shown to be necessary for inducing eye development in mice, as *Rax* knockouts do not form optic structures [29,30]. In *Xenopus*, Otx2 and Sox2 have been shown to directly synergistically activate *Rax* through binding to a non-coding sequence 2 kb upstream of the *Rax* promoter, which is conserved across vertebrates [28]. Furthermore, *Sox2* missense mutations identified in patients with ocular disorders were found to disrupt the interaction of Otx2 and Sox2, as well as Sox2 DNA-binding ability, perturbing the ability of Otx2 and Sox2 to regulate *Rax* expression [28]. The role of *Six3* in early eye development has been shown in mice, where Six3 is required for repression of *Wnt1* transcription, which is necessary for regionalisation of the forebrain, as *Six3*-/- mice show expansion of *Wnt1* expression, truncated rostral forebrain and lack eyes [3,31]. EFTFs form a self-regulating network which are sufficient to coordinate the development of the eye through suppression of genes which antagonise eye development, such as *WNT*, and upregulation of genes required for eye development, such as *BMP4*, demonstrated in *Xenopus* by the ability of EFTF RNA cocktails to induce ectopic eye formation [1,2,4,25,32,33]. *OTX2* is subsequently downregulated in the eye field [2,24].

### 2.2. Splitting of the Eye Field

At 22 days gestation, *SIX3*-regulated *SHH* establishes the midline and factors from TGFβ, FGF and SHH families secreted from the underlying axial mesoderm stimulate the anterior migration of prospective hypothalamic cells, causing the eye field to split in two (Figure 2c) [1,2,3,24]. The direct regulation of *Shh* by *Six3* has been observed in mice, and loss of *Shh* signalling in mice leads to defective midline patterning, including cyclopia, also found in humans with mutations in *SIX3* and *SHH* [1,3,34,35]. Experiments using medaka fish show that FGF signalling is required for neural patterning to establish a competence region for *Shh* signalling, and FGFs interact with Shh to activate downstream targets of *Shh* required for optic development [2,36]. *Ndr2*, a member of the TGFβ family, is required for midline signalling in zebrafish, and loss of *Ndr2* expression leads to cyclopia [2,3,37]. Tracking the movement of neural cells in zebrafish has shown that the single eye field is split through the anterior migration of cells, and cells in *Ndr2* mutant zebrafish fail to migrate, resulting in a single cyclopic eye [2,38]. *RAX*-positive cells of the eye field proliferate and migrate bilaterally, resulting in evagination of the optic primordia from the forebrain and the generation of bilateral optic pits/grooves, observed through fluorescent tagging of *Rx3* in wild-type and mutant medaka fish (Figure 2d,e) [2,4,24,25,30,39,40]. From 22–27 days gestation, further evagination of the optic pits through the cephalic mesenchyme results in the formation of optic vesicles (Figure 2e,f) [2,3].

### 2.3. Lens Placode Formation

**Figure 3:**
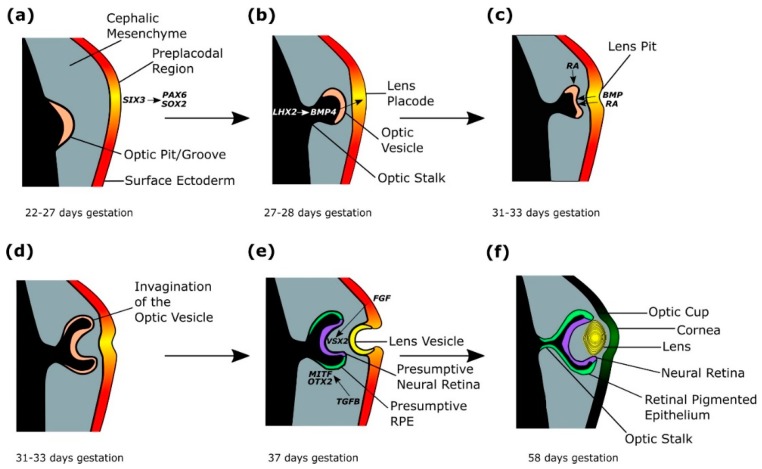
The genetics of optic cup and lens formation. (**a**) A preplacodal region develops within the surface ectoderm overlying the optic pit/groove, stimulated by expression of *SIX3*, which activates *PAX6* and *SOX2*. (**b**) The lens placode is signalled to thicken through *LHX2*-regulated *BMP4* expression from the evaginating optic vesicle. (**c**) The developing lens placode releases BMP and retinoic acid (RA), which bind to the optic vesicle, stimulating coordinated invagination of the optic vesicle and lens placode, which forms the lens pit. (**d**) The invagination of the optic vesicle results in the formation of a bilayered optic cup. (**e**) TGFβ signalling from the extraocular mesenchyme induces and maintains *MITF* and *OTX2* expression in the outer layer of the optic cup, which forms the presumptive retinal pigmented epithelium (RPE). *FGF* signals from the surface ectoderm stimulate the inner layer of the optic cup to form a *VSX2*-expressing presumptive neural retina (NR). The continued invagination of the lens pit results in the formation of the lens vesicle. (**f**) From 47 days gestation, retinal differentiation occurs, with the inner layer of the optic cup forming the neural retina (NR) and the outer layer forming the retinal pigmented epithelium (RPE). The lens vesicle detaches from the surface ectoderm and forms a definitive lens by 58 days gestation.

Within the overlying surface ectoderm, expression of *SIX3* directly activates *PAX6* and *SOX2*, stimulating the formation of a pre-placodal region from 22–27 days gestation (Figure 3a) [4,41,42,43]. *Six3* regulation of the lens pre-placodal region has been demonstrated by conditional deletion of *Six3* in mice, leading to downregulation of *Pax6* and loss of *Sox2* expression, as well as failure of the pre-placodal region to thicken [4,42]. Furthermore, ChIP, EMSA and luciferase reporter assays, show that *Six3* activates *Pax6* and *Sox2* expression in mice [42]. *LHX2*-regulated *BMP4* expression from the evaginating optic vesicle signals the thickening of the lens placode at day 27–28 of gestation (Figure 3b) [1,3,4,41,43,44,45]. The importance of *Bmp4* in lens induction has been shown in mouse models, where homozygous mutant mice lack lenses, but the phenotype can be rescued by exogenous BMP4 protein [45]. *Lhx2* has been shown to regulate *Bmp4* expression in the optic vesicle of mice, as *Lhx2*-/-mice have downregulated *Bmp4* in the optic vesicle, and failure of lens formation [43,46].

### 2.4. Optic Cup Formation

The developing lens placode releases BMP and retinoic acid which bind to the optic vesicle, stimulating invagination of the optic vesicle from 31–33 days gestation (Figure 3c,d) [1,4,47,48,49,50,51,52]. Simultaneously, retinoic acid signalling from the surrounding periocular mesenchyme aids invagination of the optic vesicle (Figure 3c) [48,51]. Retinoic acid requirement in optic cup formation has been studied in mouse models, where double knockouts of retinoic acid synthesising retinaldehyde dehydrogenases developed optic vesicles that failed to invaginate, which were rescued by maternal dietary retinoic acid administration [1,4,47,48,49,50,51,52]. Experiments in mice show that filopodia extending mostly from the lens ectoderm tether the lens to the presumptive retina, allowing coordinated invagination of the presumptive lens and optic vesicle, and loss of filopodia in mutant mouse models results in reduced lens pit invagination (Figure 3c–e) [4,53]. In mice and chicks, lens invagination has been shown to occur through apical constriction, but the mechanisms driving optic vesicle invagination are not entirely clear, although experiments disrupting the extracellular matrix in chick embryos, alongside computational investigation have shown the extracellular matrix is required for proper formation and invagination of the lens placode and optic vesicle, through spatial restriction of proliferating cells [54,55,56,57].

The invagination of the optic vesicle results in the formation of a bilayered optic cup (Figure 3d,e) [33]. TGFβ signalling from the extraocular mesenchyme induces and maintains *MITF* and *OTX2* expression in the outer layer of the optic cup, which forms the presumptive retinal pigmented epithelium (RPE), demonstrated experimentally through the removal of the extraocular mesenchyme in developing chick eyes resulting in absent RPE, which was restored through addition of the TGFβ family member activin (Figure 3e,f) [4,24,44,58,59,60,61]. *MITF* and *OTX2* in the distil optic vesicle activate RPE differentiation genes, such as *QNR71*, *Tyr*, *Trp1* and *Trp2* in mice [24,62]. Reduction of *MITF* signalling in a human embryonic stem cell (hESC) model of eye development disrupted RPE maturation [61]. Additionally, *Otx1* and *Otx2* mutant mouse models show loss of RPE-specific *Mitf* expression, and expansion of neural retina (NR)/optic stalk regions [63]. FGF signals from the surface ectoderm stimulate the inner layer of the optic cup to form *VSX2*-expressing presumptive NR, shown in chick development through *Fgf8* localisation to the neural retina, and in vivo application of FGF8, inducing presumptive chick RPE to differentiate into ectopic NR through preventing expression of RPE marker *Mitf*, and inducing NR markers *Rx* and *Sgx1* (Figure 3e,f) [3,4,24,64]. *VSX2* in the proximal optic vesicle represses RPE marker *MITF*, and is required for cell proliferation [24,65,66,67,68]. In *Vsx2* mutant mice, *Mitf* is ectopically expressed in the NR, indicating *Vsx2* represses *Mitf* expression in the NR [24,68]. Furthermore, mice with impaired *Vsx2* production have a small eye phenotype due to decreased proliferation of retinal progenitor cells [24,69]. *PAX6* expression in the presumptive NR maintains multipotency in retinal progenitor cells, allowing subsequent differentiation into the seven retinal types: retinal ganglion cells, horizontal cells, cone photoreceptors, rod photoreceptors, amacrine cells, bipolar cells and Müller glia [24,70]. Inactivation of *Pax6* in mice retinal progenitor cells using conditional gene targeting restricts the cell fate of retinal progenitor cells to amacrine cells [24,70].

The optic cup is patterned through antagonistic expression of *PAX2* and *PAX6*, shown in mouse models as *Pax6*-/- mutants show expansion of the *Pax2* expression domain into the optic stalk, and *Pax2*-/- mutants show expansion of the *Pax6* expression domain into the optic cup, and analysis of the molecular interactions of *Pax6* and *Pax2* in mice show reciprocal transcriptional repression [24,32,71,72]. *SHH*-regulated *VAX1/VAX2* expression induces *PAX2* expression and represses *PAX6* expression in the proximal optic vesicle, inducing optic stalk formation, demonstrated experimentally by injection of *Shh* mRNA into *Xenopus* embryos, resulting in expansion of the *Vax1* and *Pax2* domain, at the expense of the *Pax6* domain, in addition to abrogation of *Vax1*/*Vax2* in zebrafish, leading to the expansion of retinal tissue into the optic nerve territory [24,32,73,74,75]. The optic fissure forms on the ventral surface of the optic vesicle from four weeks gestation to enable the hyaloid vasculature to enter and supply the developing eye with nutrients [76]. This opening fuses by week seven of gestation through apposition of the epithelial margins around the vasculature, spatial specification along the proximal-distal axis of the fissure and basement membrane breakdown, resulting in the formation of a continuous epithelial layer [77,78,79].

## 3. The Genetic Basis of Anophthalmia and Microphthalmia

AM has a complex aetiology, with dominant, recessive, autosomal and X-linked inheritance, although most mutations associated with non-syndromic AM arise de novo sporadic [11,12]. Non-penetrance has been observed, where family members carrying the same variant as the AM proband exhibit no ocular phenotype, as well as variable expressivity, where relatives with the same variant exhibit a spectrum of AM and related ocular features [80,81]. Variable expressivity and non-penetrance may be due to genetic modifiers, and/or stochastic environmental factors which influence the levels of gene expression [80]. Some cases of AM have been shown to arise through parental germline mosaicism, where a parent expresses no ocular phenotype as the mutation is not present in the ocular-derived tissue, but is present in gonadal tissue due to postzygotic mutational events [10,80,82,83,84]. This complex pattern of inheritance has implications for providing meaningful genetic counselling [85]. Chromosomal abnormalities, including aneuploidy, triploidy, translocations, deletions and duplications account for 20%–30% of AM [9,10,85,86]. Over 90 individual genes are associated with AM (Table 1), most of which encode transcription factors involved in eye morphogenesis, or components of the retinoic acid signalling pathway [12,87,88]. Pathogenic monogenic mutations include missense, nonsense, deletions, insertions and splice-site variants [12,66].

There is significant inter- and intra-familial phenotypic variability amongst patients and this may be related to specific genotype–phenotype correlations, or yet unidentified genetic modifiers. In *PAX6*-related ocular disease, haploinsufficiency gives rise to a range of phenotypes; the majority of loss-of-function alleles result in classic aniridia (with full iris and foveal hypoplasia, nystagmus, cataract, glaucoma and corneal keratopathy [211]) and missense mutations in the DNA-binding paired domain result in a milder phenotype, such as dominant nystagmus with foveal hypoplasia and normal irides [212]. Interestingly, a recent report highlights recurrent missense variants, p.S54R and p.N124K, associated with severe bilateral microphthalmia, phenocopying *SOX2*-associated AM [213]. An electromobility gel-shift assay (EMSA) revealed that for p.S54R, an 85% reduction in binding to the PAX6 protein-responsive element termed the LE9 enhancer was seen compared to wild-type protein, but with no effect on binding to the SIMO element (an autoregulatory PAX6 binding site with enhancer activity, resulting in correct maintenance of *PAX6* expression). The p.N124K variant showed a moderate reduction in binding to both DNA enhancer sequences. The precise location of variants can affect protein function, and the lack of established genotype–phenotype relationships highlight the need for more correlation studies in disorders with Mendelian inheritance and phenotypic heterogeneity, such as AM. This will guide clinicians on prognosis and identifying potential therapeutic targets [85]. Furthermore, exploring the genetic pathways frequently disrupted in AM will support the identification of novel causative genes, variants, and regulatory elements which may act as genetic modifiers [85]. The more common genes associated with AM, with evidence from animal and cellular modelling, will be outlined below. The main genes fall into two distinct categories (i) eye field initiating transcription factors, such as *SOX2*, *OTX2*, *RAX*, *VSX2* and *PAX6* or (ii) retinoic acid signalling pathway components, including *STRA6*, *ALDH1A3* and *RARβ* [12,87,88].

### 3.1. Eye Field Initiating Transcription Factors

#### 3.1.1. SOX2

Heterozygous variants of sex-determining region Y-box 2 (*SOX2*) are the most common known cause of bilateral anophthalmia and severe microphthalmia, accounting for 15%–40% of cases [8,12,19]. *SOX2* mutations are associated with isolated unilateral or bilateral AM, complex AM, and Syndromic Microphthalmia 3 (MCOPS3), where extraocular features include brain anomalies, neurocognitive delays, seizures, sensorineural hearing loss, oesophageal atresia, short stature, microcephaly and genital anomalies (Table 1) [8,12,89]. Over 76 disease-associated variants of *SOX2* have been identified, incorporating loss-of-function deletion, missense, frameshift and nonsense mutations [7,8,12,19]. The majority of AM pathogenic *SOX2* mutations arise de novo (60%), but alleles can also be inherited in an autosomal dominant manner, causing AM due to *SOX2* haploinsufficiency [7,8,12,89]. Disease-associated *SOX2* variants have been inherited from asymptomatic parents as a result of mosaicism, such as a case of a mother with no ocular features who had a daughter with bilateral anophthalmia along with extraocular features of *SOX2* syndrome as a result of germinal mosaicism [83,84]. Consequently, it is important to screen parents for accurate genetic counselling and future family planning [6,9,10,11].

SOX2 is a high-mobility group transcription factor [8,28]. In *Xenopus*, Sox2 and Otx2 have been shown to function together to coordinate *Rax* expression in the early eye field [28,29]. Furthermore, introduction of point mutations in *Xenopus* Sox2 protein (to replicate missense mutations found in human ocular abnormalities) resulted in disrupted *Rax* transactivation and reduced DNA and Otx2-binding ability of Sox2 [28]. These findings indicate a key role for Sox2 in early eye formation, through coordinated regulation of EFTF *Rax* in conjunction with Otx2, and consequently, disruption of Sox2 results in disrupted eye development through perturbation of the Rax signalling network [28,29]. The severity of ocular abnormalities observed in humans indicate the role of SOX2 may reflect that of *Xenopus*, with a conserved key function in transcriptional regulation of key genes in human early eye development.

A genotype–phenotype correlation has been suggested for *SOX2* mutations in AM cohorts, as missense mutations, particularly in the DNA-binding or transactivation domains, often result in non-penetrance or a milder ocular phenotype, such as ocular coloboma, and fewer developmental and systemic abnormalities, including postnatal growth retardation [8,10,12]. However, a clear correlation has not been definitively proved [8]. Variable *Sox2* ocular phenotypes have been observed in a gene-dosage allelic series of mouse models, which displayed a range of eye phenotypes where the severity correlated to the expression of *Sox2*, suggesting a relationship between *SOX2* genotype and clinical phenotype in humans may exist, due to dosage sensitivity in eye development [10,214].

#### 3.1.2. OTX2

Between 2%–8% of AM cases are estimated to be the result of a pathogenic mutation in *OTX2* [8,9,18,90,91]. Combined, *SOX2* and *OTX2* variants account for at least 60% of bilateral severe cases of AM [12,19]. Variants of *OTX2* include indel, missense, frameshift and nonsense mutations, and AM is usually the result of a heterozygous loss-of-function [8,12,19,91]. While 40% of *OTX2* mutations arise de novo, 35% are inherited from an unaffected parent, which complicates inheritance patterns [8,12,19,91]. Frequency of non-penetrance is high, with carrier parents displaying no ocular phenotype, as well as variable expressivity, for example, a case where one patient displayed unilateral microphthalmia, and his brother had a right anophthalmic eye and left ocular coloboma [12,18,19,80,91]. This case was believed to be a result of gonosomal mosaicism, as neither parent was found to carry the mutation in blood or buccal cell DNA [18]. Mosaicism has been confirmed in other *OTX2* cases, such as a patient with bilateral severe microphthalmia who was the daughter of a phenotypically normal mother shown to have low levels of the variant in blood and buccal DNA, indicating gonosomal mosaicism [12,18,19,80,91]. *OTX2* patients encompass extremely variable phenotypes, including additional eye malformations such as anterior segment dysgenesis, retinal dystrophy and hypoplasia or aplasia of the optic nerve and optic chiasm, as well as syndromic features including pituitary abnormalities, hypopituitarism, brain anomalies, seizures and developmental delay (Table 1) [8,91,93,215]. Homozygous mouse knockouts of homeobox gene *Otx2* lack eyes, while heterozygous knockout mouse models also show a highly variable phenotype, from acephaly, micrognathia, anophthalmia, microphthalmia to normal development, depending on the genetic background [8,25,63,80,215,216,217,218,219]. A relationship between genotype and clinical phenotype has been observed, as mutations in the second half of *OTX2* seem to occur more frequently with abnormal pituitary function, although this correlation is not always seen [8,91,215].

*OTX2* is required for neural development, and coordinates the expression of EFTFs *SIX3*, *RAX* and *PAX6* to initiate eye field development, but is subsequently downregulated in the eye field by EFTFs including *RAX* [3,25,32,220,221,222]. *Otx2* has been shown to be necessary for eye development in mouse and *Xenopus*, as early EFTFs *Six3* and *Pax6* only possesses the ability to induce ectopic eyes in the presence of *Otx2* expression [25,26,27,63,223]. Furthermore, overexpression of *Otx2* in *Xenopus* results in upregulation of *Rax*, indicating *Otx2* is a positive regulator of *Rax* in early development [28].

*OTX2* also plays a role later in eye development, during the regionalisation of the optic vesicle, where TGFβ signalling from the extraocular mesenchyme induces *OTX2* expression in the distil optic vesicle [24]. *WNT* signalling then induces upregulation of *OTX2* in the prospective RPE [24]. *OTX2* is required for RPE maintenance and differentiation, including regulation of key RPE marker *MITF* [24,63,224]. Inactivation of *Wnt* signalling activator *β-catenin* in mice at the optic vesicle stage results in a lack of *Otx2* and *Mitf* signalling in the optic cup, and expansion of the NR domain, leading to anophthalmia [24,59].

#### 3.1.3. RAX

Biallelic mutations in the EFTF *RAX* are estimated to account for 2%–3% of AM [6,7,11,94]. Disease-associated loss-of-function variants of *RAX* include missense, nonsense, frameshift and splicing mutations, as well as whole gene deletions [6,12]. Heterozygous carriers of disease-associated variants display no ocular phenotype, indicating an autosomal recessive inheritance pattern [6,7,12]. Homozygous or compound heterozygous *RAX* variants are often associated with bilateral severe AM, alongside neurological features such as intellectual deficiency and autism [7].

*RAX* has been shown to play a conserved role in establishment/proliferation of the eye field, as expression of ectopic *Rax* in *Xenopus* results in formation of ectopic retinal tissue [1,30], and zebrafish/mouse heterozygous knockouts of *Rax* have an eyeless phenotype [30,225,226,227]. The homeobox gene *Rax* is expressed early in eye development, and has been identified as necessary for the initiation of eye field development through regulation by *Otx2* and EFTFs *Six3* and *Pax6* in targeted mouse knockout models [228]. *Rax* has been shown in zebrafish to coordinate the splitting of the eyefield, by downregulating *nlcam* in the eye field, to decelerate the convergence of retinal progenitor cells to the midline of the neural plate [39,40]. Overexpression of *nlcam* results in abnormal migration of the eye field, converging further towards the midline, resulting in a small eye phenotype in zebrafish [39]. Furthermore, in chimeric mouse embryos of *Rax*-/- and wild-type cells, *Rax*-deficient cells were not incorporated into optic tissue, indicating a role for *Rax* in cellular segregation in the early developing eye [4,29].

#### 3.1.4. VSX2

Biallelic mutations in *VSX2* are thought to account for 2% of isolated microphthalmia, and were identified in 1.5% of all MAC (microphthalmia, anophthalmia and coloboma) cases in a screening of >880 samples [9,12]. Disease-associated variants include missense, nonsense, exon deletions and splice-site variants [66,95]. *VSX2* has an autosomal recessive inheritance pattern, and homozygous or compound heterozygous *VSX2* variants are usually associated with bilateral AM [12]. *VSX2* variants can result in isolated AM, although complex cases often include cataracts, as well as iris abnormalities, retinal dystrophy and colobomas, and individual cases with systemic features have been reported, including developmental delay with behavioural problems, autism, cryptorchidism, ovarian defects, or hearing impairment, although no genotype–phenotype correlations have been identified [12,17].

*VSX2* is a homeodomain-containing transcription factor [95]. Inactivation of the *VSX2* orthologue *Alx1* in zebrafish results in small eyes [95,229]. *Vsx2* null mice are microphthalmic, and show reduced proliferation of retinal progenitor cells, as well as transdifferentiation of the neural retina into RPE [69,95,230,231]. *Vsx2* has been shown to regulate retinal cell number via *CycD1*-mediated repression of the negative regulator of proliferation cyclin-dependent kinase inhibitor *Kip1*, in retinal progenitor cells [231]. Consequently, *Vsx2* enables cell proliferation by preventing retinal progenitor cells from exiting the cell cycle [66,231]. This has been shown as in *Vsx2* null mice, *Kip1* is abnormally present in retinal progenitor cells, resulting in cell number decrease in the retina and a small eye phenotype [231].

In the optic vesicle, *VSX2* expression marks the developing neural retina, while *MITF* is expressed in the developing RPE [32]. *Fgf* expression activates *Vsx2* in mouse neural retina which represses *Mitf* transcription, and *Vsx2* null mice show erroneous expression of *Mitf* in the neural retina [66,68]. Analysis of the function of *VSX2* in human iPSC-derived optic vesicles showed *WNT* to be a downstream target [232]. Patient-derived iPSCs with a mutation in *VSX2* found upregulation of WNT pathway components and RPE markers, demonstrating *VSX2*-mediated repression of *WNT* contributes to maintaining neural retina identity [232].

#### 3.1.5. PAX6

*PAX6* is a transcription factor most commonly associated with aniridia [92,233]. A study of >500 MAC cases found 1%–2% to be *PAX6* related [12]. Heterozygous loss-of-function *PAX6* variants have been found in patients with unilateral and bilateral AM, indicating an autosomal dominant inheritance pattern [7,12,92]. Patients with *PAX6-*associated microphthalmia often have a complex phenotype, with associated ocular abnormalities including aniridia, iris hypoplasia, coloboma and optic nerve hypoplasia (Table 1) [12]. Mosaicism has been reported in *PAX6-*associated microphthalmia, such as a case with two brothers with bilateral complex microphthalmia with a heterozygous missense mutation, and an unaffected mosaic mother [92].

Missense mutations in the paired-box domain of *PAX6* have been more frequently been associated with MAC, which is the domain known to interact with SOX2 [12,213,234]. The complex formed by PAX6 and SOX2 is known to be key for initiation of lens development by activation of crystallin genes [3,234]. Correct formation of the lens is vital to optic cup development, indicating a potential pathway for *PAX6* mutations in the paired-box domain to have an increased likelihood to cause AM.

The homeobox gene *PAX6* has been shown to be a conserved regulator of eye development [42,235]. *Pax6* mutant mouse models display small eyes alongside other ocular phenotypes [92,236,237]. *Pax6-/-* mice develop eyes, but they fail to be maintained, arresting in development prior to the optic cup stage [2,236,237,238]. *PAX6* is one of the first EFTFs to be expressed, and ectopic expression of *Pax6* in *Xenopus* can be sufficient to induce ectopic eye tissue [223,235]. Furthermore, misexpression of *Pax6* in *Xenopus* results in erroneous upregulation of EFTFs including *Six3*, *Rax* and *Lhx2* [25,223,235]. *Pax6* is known to form functional complexes with *Lxh2* in mice, which are required for the regulation of *Six6* in the optic vesicle [235,238]. These results indicate *PAX6* plays a conserved key role in initial eye formation by initiating a cascade of gene expression which regulates eye development [223].

*Rax*-deficient mice show loss of *Pax6* expression in the region of the neural plate which would form the optic primordium, suggesting *Rax* is an upstream regulator of *Pax6* in early eye field formation [32,228]. In *Six3* mutant mice, *Pax6* is downregulated in the preplacodal lens ectoderm, indicating *Pax6* in the developing lens is also activated by *Six3* [26,42]. *Bmp7* has also been implicated in the initiation of *Pax6* in lens induction, as in *Bmp7* null mice, *Pax6* expression is lost in the lens placode, resulting in lens defects [3,239].

Moreover, *PAX6* is required for the dorso-ventral patterning of the optic cup, where *PAX6* is highly expressed in the dorsal region and *PAX2* is expressed in the ventral region [3,235]. In *Pax6* mutant mice, there is dorsal expansion of *Pax2* expression, while in *Pax2* mutants there is ventral expansion of *Pax6*, demonstrating the antagonistic function of *Pax6* and *Pax2* in the optic cup [3,72]. In addition, this patterning is regulated in mice by *Shh* which promotes ventral expression of *Vax1* and *Pax2*, while inhibiting expression of *Pax6* and *Rax* [24,73,235,240]. In *Vax1/Vax2* double-mutant mice, the optic nerve is transformed into retinal tissue, and *Pax6* expression is increased in the ventral region of the optic cup, demonstrating the importance of *Vax1/Vax2* in regulating *Pax6* expression in the optic cup [75]. *Lhx2* mutant mouse optic vesicles show lack of downstream target *Vsx2* and display expansion of *Pax6* ventrally, indicating *Pax6* is partially regulated in the optic cup through *Lhx2*-mediated *Vsx2* expression [32,46].

### 3.2. Retinoic Acid Signalling Pathway Components

#### 3.2.1. STRA6

Biallelic loss-of-function mutations in *STRA6*, including missense, nonsense, frameshift and whole gene deletions, can lead to AM, usually manifesting bilaterally [8,12,97,241]. Commonly, pathogenic recessive *STRA6* variants result in Microphthalmia, Syndromic 9 (MCOPS9), also known as Matthew Wood Syndrome, where syndromic features include pulmonary, diaphragmatic and cardiac defects, resulting in death within the first 2 years of life [12,17,96,242]. However, milder phenotypes have been observed, including a patient with bilateral anophthalmia and mild syndromic features, in addition to isolated AM in patients with homozygous missense *STRA6* variants [19,20]. However, no genotype–phenotype correlation has yet been established [8,96,98].

*STRA6* is a transmembrane receptor for retinol-binding protein, responsible for mediating vitamin A uptake into cells [8,20,98,243,244]. Cellular analysis showed that a missense mutation in *STRA6* resulted in loss of vitamin A uptake [20]. Furthermore, the clinical phenotype is recapitulated in zebrafish models, with inhibition of retinoic acid resulting in microphthalmia, and developmental defects including heart morphogenesis [20]. Patients with *STRA6* variants display variation in phenotype, such as one family where phenotype ranged from bilateral anophthalmia to unilateral microphthalmia with contralateral ocular coloboma, indicating phenotypic variation may result from environmental factors such as variable vitamin A uptake, as well as genetic background [8,20,98].

The severity of phenotypes associated with *STRA6* variants demonstrate the importance of retinoic acid signalling in development [98]. Retinoids can regulate development through enhancing or suppressing developmental gene expression [20,245,246]. In eye organogenesis, retinoic acid is required to signal the formation of the lens placode, in addition to the invagination of the optic vesicle [43,48,49]. Absence of *Stra6* in zebrafish impairs retinoic acid receptor signalling and gene regulation, resulting in craniofacial and cardiac developmental defects, as well as microphthalmia [245]. The results demonstrated in animal and cellular modelling indicate a conserved function of *STRA6* in mediating vitamin A uptake, and that disruption of *STRA6* results in AM due to disruption of the retinoic acid signalling pathway, and consequently, disrupted expression of genes required for eye development, including lens placode development and optic cup invagination [245].

#### 3.2.2. RARβ

AM is associated with recessive loss-of-function or dominant gain-of-function pathogenic variants of *RARβ* [8,21,87,88]. Variants include missense, nonsense and frameshift mutations [87]. Gain-of-function missense *RARβ* variants found in AM patients were shown to exhibit increased response to retinoic acid ligands compared to the wild-type, indicating an increase in retinoic acid signalling mediated by *RARβ* is sufficient to disrupt eye development [87,88]. The clinical manifestation of *RARβ* mutations resemble those of *STRA6*, including pulmonary and cardiac defects, often resulting in termination of pregnancy or neonatal death [8,12,87,88]. 

*RARβ* is one of three retinoic acid receptors, with *RARα* and *RARγ*, which transduce retinoic acid signalling to allow retinoic acid to regulate specific target genes in development [51,88]. The retinoic acid receptors form heterodimers which are key to oculogenesis [48,51]. A mouse model with all three retinoic acid receptors inactivated resulted in clinical anophthalmia, including lack of optic nerve [51]. In mouse models, *Rarβ*-mediated retinoic acid signalling in the periocular mesenchyme has been shown to regulate apoptosis and the expression of *Foxc1* and *Pitx2*, which coordinate the development of the anterior eye segment [51,247,248].

#### 3.2.3. ALDH1A3

Mutations in the retinaldehyde dehydrogenase gene *ALDH1A3* are associated with an estimated 10% of AM [21,99]. Disease-associated variants include missense, nonsense and exon skipping [99,100,101,249]. Biallelic *ALDH1A3* mutations tend to be associated with bilateral AM, and can be isolated or complex (Table 1) [12]. Biallelic pathogenic AM *ALDH1A3* alleles are commonly associated with a neurocognitive phenotype, but additional extraocular findings are rare [8,12,99]. Non-penetrance has been reported with *ALDH1A3* disease-associated variants, such as a case of autosomal recessive *ALDH1A3* variants where an asymptomatic sister carried the same homozygous mutation as two siblings affected with bilateral AM [101,249].

*ALDH1A3* is part of the retinoic acid synthesis pathway, through oxidation of retinaldehyde to retinoic acid [8,48,99]. *ALDH1A3* is one of three human retinaldehyde dehydrogenases, which have specific spatial and temporal expression patterns [48,50,250]. *Pax6* mutant rat embryos are deficient in retinaldehyde dehydrogenase *Raldh3* (Human *ALDH1A3*), indicating *Raldh3* is genetically regulated by key oculogenesis transcription factor *Pax6* [48,251].

The mouse retinaldehyde dehydrogenase gene *Raldh2* (human *ALDH1A2*) is expressed in the periocular mesenchyme [50,250]. Loss-of-function mutations in *Raldh2* inhibit optic cup invagination [49]. Consequently, *ALDH1A2* in humans is thought to regulate retinoic acid synthesis signalling to the invaginating optic vesicle through signalling from the periocular mesenchyme [48]. However, in *Raldh1-/-*(Human *ALDH1A1*)*/Raldh2-/-* mouse embryos, retinoic acid synthesised by *Raldh3* in the RPE acts on the neural retina to aid ventral invagination [48,50,250]. *Raldh3*-/- mouse mutant embryos initiate optic cup formation, but lack retinoic acid in the ventral optic cup, resulting in ventral retina shortening [250,252]. Consequently, there appears to be a conserved role for *ALDH1A3* in synthesis of retinoic acid in the developing ventral optic cup, with mutations resulting in defects in optic cup formation, including invagination [48,250].

## 4. Current Research and Future Directions

### 4.1. Modelling Eye Development Using iPSCs

Current understanding of eye development is heavily based on evidence from animal modelling, focusing on zebrafish, *Xenopus* and mouse [76,253,254]. While conserved molecular pathways indicate a fundamental similarity across vertebrate eye development, conflicting evidence from experiments performed on different model organisms indicates a degree of divergence in the morphogenesis of the eye [255,256]. For example, the development of the cornea is known to vary by species; stratification of the human corneal epithelium occurs early in development, resembling chick development, while the development of the mouse corneal epithelium does not fully stratify until 10 weeks after birth [255]. Furthermore, the migration of neural crest cells which form the corneal endothelium and stromal keratocytes occurs in two waves in reptiles, birds and primates, while in rodents, cats, rabbits and cattle, this occurs in a single wave of cell migration [256]. To comprehensively understand the molecular basis of human developmental eye defects and ultimately, develop functionally viable therapies, it is vital to obtain an accurate understanding of human eye development, and appreciate how it differs from that of model organisms.

One method for understanding human biology is the use of human induced pluripotent stem cells (hiPSCs) [257,258]. Long-term modelling of hiPSCs can enhance our understanding of normal human cellular behaviour, as well as better comprehending diseases by deriving patient iPSCs which can be used for investigating the basis of phenotypic heterogeneity, in addition to therapy screening and personalised medicine [257,259]. Current work in developing methodologies for organoid modelling of eye development show potential for gaining a working understanding of human eye morphogenesis, and developing therapies [259,260]. RNA-seq transcriptome analysis indicates that stem-cell derived optic cups recapitulate human eye development in vivo, through temporal expression of cell differentiation markers and retinal disease genes, as well as mRNA alternative splicing [261,262,263]. Early-stage optic vesicle-like structures generated from hiPSCs derived from a microphthalmic patient with a *VSX2* null variant showed upregulation of WNT pathway components and RPE marker, *MITF*, which were rescued by pharmacological inhibition of *WNT* signalling [232]. These results showed a key role for *VSX2* in *WNT* signalling and maintenance of the neural retina through WNT pathway suppression [232]. This study shows the potential utility of hiPSC modelling in improving understanding of human eye development, and developing novel therapies. However, a constraint of in vitro modelling is the lack of vasculature and surrounding tissues, which may result in loss of vital external cues for recapitulating in vivo development [264]. Additionally, whole organism modelling is necessary for understanding the effect of gene activity on organogenesis of multiple organs, which is particularly important when studying syndromic disorders [265]. Utilising knowledge gleaned from in vivo animal modelling in conjunction with human cellular modelling is key to obtaining a complete picture of the molecular basis of human eye development and the role of perturbed genetic pathways in congenital disorders, such as AM.

### 4.2. Genomics and Epigenetics

The functionality and availability of genetic tools has progressed rapidly in recent years. Next-generation sequencing is a powerful tool for understanding development and disease, allowing relatively cheap, high-throughput methods for analysing the genome, transcriptome and epigenome [266]. Whole exome/genome sequencing can identify novel disease-associated coding and non-coding variants, as well as be a useful tool in diagnosing patients with heterogeneous ocular disorders with numerous underlying genetic causes, such as AM (Table 1) [81,267]. Increasing the proportion of known variants associated with AM will improve the frequency of genetic diagnosis, which is important for directing disease management, including screening for potential syndromic features, as well as providing reliable genetic counselling and family planning [11,85]. RNA-seq has aided in characterising gene expression in the developing retina, as well as elucidating genetic pathways affected in ocular disorders through differential transcriptomic analysis, such as the role of *VSX2* in regulation of the WNT pathway in retinal organoid differentiation [232,268,269]. Improved understanding of temporal gene expression throughout eye development will aid in identifying potential disease-associated variants, as well as putative therapy targets.

A potential source of disruption to the molecular regulation of the developmental pathway is epigenetic disturbance. Epigenetic changes, such as chromatin remodelling and histone modification, result in altered gene regulation, and therefore, have a great potential for disrupting the ocular developmental pathway [270,271]. Heterogeneity in epigenetic patterning in stem cell cultures has been shown to impact the ability of cells to differentiate into retinae, providing reasonable evidence that epigenetic regulation is important to normal eye development, and consequentially, may play a role in ocular malformations [272]. Epigenetic changes altering the secondary structure of DNA have been shown to induce neurodevelopmental disorders including autism [273]. Utilising epigenetic profiling techniques such as CHIP-seq to identify epigenetic variation associated with developmental ocular disorders will provide a better understanding of how these disorders arise, in addition to providing a new avenue of putative treatments to explore [266,274,275].

### 4.3. Proteomics and Metabolomics

Post-translational modifications can have a significant impact on protein activity, and consequently, cellular function, for example, post-translational modifications play a key role in the regulation of zebrafish retina regeneration [276,277,278]. Consequently, investigating the proteome alongside the transcriptome is key to understanding cellular behaviour in eye development. Additionally, the impact of downstream metabolites in temporal mediation of cellular behaviour has been revealed in recent years [279]. Advances in metabolomic investigation, such as metabolome profiling, will provide greater insight into different aspects of cellular processes and how metabolites impact development, in addition to providing new biomarkers for understanding the pathways disrupted in disease and targets for neonatal screening therapies [280,281].

### 4.4. Therapeutics

One of the fundamental reasons for investigating the molecular basis of human eye development and disease is to manipulate the knowledge gained into targeted therapies that will benefit patients. As AM is a structural eye defect arising from three weeks gestation, developing prenatal treatments has been difficult as the mother may not know they are pregnant and compounds may have an adverse effect on both the mother or the rest of the developing foetus. However, with the first in utero exploratory multicentre phase I/II clinical trial (NCT03706482) evaluating safety and efficacy of postnatal or prenatal intravenous administration of allogeneic expanded foetal mesenchymal stem cells for the treatment of severe osteogenesis imperfecta underway, there may be potential applicability for congenital ocular disorders in the future.

Small-molecule drugs, such as Ataluren, target in-frame nonsense mutations by interfering with ribosomal fidelity to enable near-cognate amino acids to be substituted rather than a release factor, thus enabling the production of full-length functional proteins. This drug had positive results as a postnatal treatment of aniridia in mouse models, demonstrating developmental plasticity of the eye. A phase 2 multicentre randomized, double-masked, placebo-controlled clinical trial (NCT02647359) is currently underway to assess the safety and efficacy of Ataluren for nonsense-mediated aniridia. If successful, this presents another avenue of post-natal treatment for certain genetic congenital diseases such as microphthalmia, where growth and visual maturation may be encouraged [282].

## 5. Conclusions

This review has highlighted the key molecular coordinators of early-eye development, and summarized current understanding of how disruption of these precise spatiotemporal regulators can lead to disruption of eye organogenesis, resulting in anophthalmia or microphthalmia (AM). Recent advances in the field of genetics, including high-throughput next-generation sequencing technology has progressed knowledge of the genetic basis of AM, as well as improving diagnosis and genetic counselling [85]. Increasing genetic screening of AM patients using whole exome/genome sequencing will continue to improve the rate of diagnosis, through identifying novel disease-associated genes and isoforms [85]. Furthermore, increased genetic diagnosis will provide larger cohorts to analyse the AM genotype/phenotype relationship, including identifying environmental factors or genetic modifiers which may result in heterogeneity and variable expressivity observed in AM [85]. Additionally, utilising animal and cellular models of AM, in conjunction with genome-editing technology such as CRISPR, will further advance understanding of function and interactions of molecular regulators of eye development, and how pathogenic alleles result in AM, with the ultimate aim of creating therapies that may aid patients with AM [85,232].

## Figures and Tables

**Table 1:** Genes associated with anophthalmia and microphthalmia. A—unilateral or bilateral anophthalmia. M—unilateral or bilateral microphthalmia. I—Isolated AM with no additional ocular features. C—complex AM (additional ocular features found in microphthalmic or contralateral eye of the patient listed). N—no syndromic features observed. S—systemic features observed.

	Gene Name	OMIM#	Disease Name	Disease OMIM#	Anophthalmia/Microphthalmia (A/M)	Isolated/Complex (I/C) (Associated Ocular Features)	Non-Syndromic/Syndromic (N/S)	Reference(s)
**1**	*SOX2*	184429	Microphthalmia, syndromic 3 (MCOPS3)	206900	A, M	I, C (coloboma, microcornea, iris defect, retinal tuft, optic nerve hypoplasia, reduced palpebral fissure, congenital cataract, glaucoma, colobomatous cyst, synechiae, anterior segment dysgenesis, retinal/chorioretinal dystrophy, myopia)	N, S	[7,10,19,89,90]
**2**	*OTX2*	600037	Microphthalmia, syndromic 5 (MCOPS5)	610125	A, M	I, C (coloboma, microcornea, retinal defect, optic nerve hypoplasia/aplasia, small/absent optic chiasm, LCA, early onset retinal dystrophy, hyperopia, amblyopia, cataract, focal retinal dysplasia, corectopia, synechiae, sclerocornea, persistent pupillary membrane, nystagmus, posterior vitreous opacity)	N, S	[7,18,19,21,80,90,91,92,93]
**3**	*RAX*	601881	Microphthalmia, isolated 3 (MCOP3)	611038	A, M	I, C (coloboma, sclerocornea, persistent fetal vasculature, retinal detachment, optic nerve atrophy/hypoplasia)	N, S	[7,90,94]
**4**	*VSX2*	142993	Microphthalmia, isolated 2 (MCOP2)	610093	A, M	I, C (coloboma, congenital cataract/cloudy cornea, iris defect, microcornea, no pupillary aperture, retinal detachment, dislocated lens, small/underdeveloped optic nerve/chiasm, retinal dysfunction)	N, S	[7,66,95]
			Microphthalmia, isolated with coloboma 3 (MCOPCB3)	610092				
**5**	*PAX6*	607108	Aniridia 1 (AN1)	106210	A, M	I, C (coloboma, aniridia/iris hypoplasia, anterior segment dysgenesis, agenesis of optic nerve/chiasm, primary aphakia, sclerocornea, congenital glaucoma)	N, S	[7,92]
**6**	*STRA6*	610745	Microphthalmia, syndromic 9 (MCOPS9)	601186	A, M	I, C (coloboma, cyst, retinal detachment, abnormal cornea/iris)	N, S	[19,20,21,96,97,98]
**7**	*RARβ*	180220	Microphthalmia, syndromic 12 (MCOPS12)	615524	A, M	I, C (coloboma, sclerocornea, anterior segment dysgenesis)	S	[21,87,88]
**8**	*ALDH1A3*	600463	Microphthalmia, isolated 8 (MCOP8)	615113	A, M	I, C (coloboma, microcornea corectopia, cyst, hypoplastic/small optic nerve/tract/chiasm, small/short palpebral fissure, conjunctival discoloration, symblepharon, nystagmus, iris attachment to the cornea)	N, S	[99,100,101]
**9**	*FOXE3*	601094	Anterior segment dysgenesis 2 (ASD2)	610256	M	C (coloboma, anterior segment dysgenesis, sclerocornea, aphakia, aniridia)	N, S	[7]
**10**	*BMP4*	112262	Microphthalmia, syndromic 6 (MCOPS6)	607932	A, M	I, C (coloboma, microcornea, retinal dystrophy, myopia, sclerocornea, anterior segment dysgenesis, corectopia, blepharophimosis, optic nerve hypoplasia, tilted/anomalous optic disc, cyst, nystagmus, cataract, glaucoma, aphakia, embryotoxon, persistent hypoplastic primary vitreous)	N, S	[102,103]
**11**	*BMP7*	112267	-	-	A, M	I, C (coloboma)	S	[104]
**12**	*GDF3*	606522	Microphthalmia, isolated 7 (MCOP7)	613704	M	I, C (coloboma, optic nerve hypoplasia, foveal hypoplasia, nystagmus)	N, S	[105]
			Microphthalmia, isolated with coloboma 6 (MCOPCB6)	613703				
**13**	*GDF6*	601147	Microphthalmia, isolated 4 (MCOP4)	613094	A, M	I, C (coloboma, optic nerve hypoplasia, foveal hypoplasia, nystagmus)	N, S	[7,21,90]
			Microphthalmia, isolated with coloboma 6, digenic (MCOPCB6)	613703				
**14**	*ABCB6*	605452	Microphthalmia, isolated with coloboma 7 (MCOPCB7)	614497	M	C (coloboma)	N	[106]
**15**	*ATOH7*	609875	Persistent hyperplastic primary vitreous, autosomal recessive (PHPVAR)	221900	M	I, C (microcornea, congenital cataract/corneal opacity, optic nerve aplasia/hypoplasia, retinal detachment/nonattachment, persistent fetal vasculature, nystagmus, vitreous degeneration, glaucoma, shallow anterior chamber, anterior displacement of the iris, peripheral anterior synechiae, calcifications present on the hyaloid membranes/retina/vitreous, vitreoretinal dysplasia)	N	[107]
**16**	*C12orf57*	615140	Temtamy syndrome (TEMTYS)	218340	M	C (coloboma)	S	[108]
**17**	*TENM3*	610083	Microphthalmia, isolated with coloboma 9 (MCOPCB9)	615145	M	C (coloboma, microcornea, nystagmus, esotropia, myopia, retinal detachment)	N, S	[109,110]
**18**	*VAX1*	604294	Microphthalmia, syndromic 11 (MCOPS11)	614402	A, M	C (optic nerve hypoplasia, small optic nerve, cyst)	S	[111]
**19**	*SMOC1*	608488	Microphthalmia with limb anomalies (MLA)	206920	A	C (optic nerve/tract aplasia)	S	[112]
**20**	*FNBP4*	615265	Microphthalmia with limb anomalies (MLA)	206920	A	I	S	[113]
**21**	*SHH*	600725	Microphthalmia, isolated with coloboma 5 (MCOPCB5)	611638	A, M	I, C (coloboma, funnel retinal detachment with sub-retinal opacity, microcornea, small optic nerve, retinal dystrophy, tilted optic disc, myopia, nystagmus, glaucoma, posterior embryotoxon)	N, S	[102,114,115]
**22**	*NAA10*	300013	Microphthalmia, syndromic 1 (MCOPS1)	309800	A	I	S	[116]
**23**	*BCOR*	300485	Microphthalmia, syndromic 2 (MCOPS2)	300166	A, M	I, C (congenital cataract, microcornea, posterior embryotoxon, secondary aphakia, secondary glaucoma, retinal detachment, persistent fetal vasculature, iris heterochromia, nystagmus, myopia, iris rubeosis, flat anterior chambers)	S	[117]
**24**	*HCCS*	300056	Linear skin defects with multiple congenital anomalies 1 (LSDMCA1)	309801	A, M	I, C (corneal opacity/cloudy and vascular cornea, cyst, sclerocornea, glaucoma,)	S	[118]
**25**	*MAB21L2*	604357	Microphthalmia, syndromic 14 (MCSKS)	615877	A, M	I, C (coloboma, microcornea, exotropia, sclerocornea, strabismus)	S	[119]
**26**	*RBP4*	180250	Microphthalmia, isolated with coloboma 10 (MCOPCB10)	616428	A, M	C (coloboma, small optic nerve/chiasm, cyst, underdeveloped extraocular muscles)	N, S	[81,120]
**27**	*GLI2*	165230	Holoprosencephaly 9 (HPE9)	610829	A, M	I, C (coloboma, optic nerve agenesis)	S	[121,122,123]
**28**	*PORCN*	300651	Focal dermal hypoplasia (FDH)	305600	A, M	I, C (coloboma, aniridia, strabismus, ectopia lentis)	S	[124,125]
**29**	*FRAS1*	607830	Fraser syndrome 1 (FRASRS1)	219000	A, M	C (fused/small palpebral fissure, cryptophthalmos)	S	[126]
**30**	*FREM1*	608944	Manitoba oculotrichoanal syndrome (MOTA)	248450	A	I, C (coloboma, obstruction of the nasolacrimal duct)	S	[127]
**31**	*SMCHD1*	614982	Bosma arhinia microphthalmia syndrome (BAMS)	603457	M	I, C (coloboma, hypertelorism, occluded or absent nasolacrimal duct, cataract)	S	[128]
**32**	*SIX6*	606326	Microphthalmia, syndromic 6 (MCOPS6)	607932	A, M	C (coloboma, cataract, nystagmus, secondary glaucoma, optic nerve dysplasia/absence of optic nerve/chiasm/tract, retinal dystrophy, cyst)	S	[129]
**33**	*TFAP2A*	107580	Branchiooculofacial syndrome (BOFS)	113620	A, M	C (coloboma, cataract/corneal clouding, reduced corneal diameter, primary aphakia, sclerocornea, retinal detachment, lacrimal duct obstruction, cyst, subluxed cataractous lens, shallow anterior chamber, persistent pupillary membrane, iris hypoplasia, dysplastic optic disc)	S	[130]
**34**	*TCTN2*	613846	Meckel syndrome, type 8 (MKS8)	613885	A, M	I	S	[131]
**35**	*CSPP1*	611654	Joubert syndrome 21 (JBTS21)	615636	A	I	S	[132]
**36**	*COL4A1*	120130	Brain small vessel disease with or without ocular anomalies (BSVD1)	175780	M	C (microcornea, Peter’s anomaly, retinal detachment, congenital cataract, glaucoma, anterior segment dysgenesis, hypermetropia, astigmatism)	S	[133,134]
**37**	*PTCH1*	601309	Holoprosencephaly 7 (HPE7)	610828	M	C (coloboma, cataract, sclerocornea, anterior segment dysgenesis)	N, S	[135]
**38**	*TBC1D32*	615867	Orofaciodigital syndrome IX (OFD9)	258865	A, M	C (coloboma)	S	[136]
**39**	*CHD7*	605806	CHARGE syndrome	214800	A, M	I, C (coloboma, microcornea, cataract, persistent fetal vasculature)	N, S	[137,138,139]
**40**	*MFRP*	606227	Microphthalmia, isolated 5 (MCOP5)	611040	M	C (retinitis pigmentosa, foveoschisis, optic disc drusen, macular edema, glaucoma, hyperopia)	N	[140]
			Nanophthalmos 2 (NN02)	609549				
**41**	*PRSS56*	613858	Microphthalmia, isolated 6 (MCOP6)	613517	M	C (hyperopia, elevated papillomacular retinal fold, shallow anterior chamber, thick lens, thickened scleral wall)	N	[141,142]
**42**	*TMEM98*	615949	Nanophthalmos 4 (NN04)	615972	M	C (hyperopia, angle closure glaucoma, narrow iridocorneal angle, shallow anterior chamber depth, optic disc drusen)	N	[143,144]
**43**	*HMGB3*	300193	Microphthalmia, syndromic 13 (MCOPS13)	300915	A, M	C (coloboma, congenital cataract)	S	[145]
**44**	*PXDN*	605158	Cornea opacification and other ocular anomalies (ASGD7)	269400	M	C (sclerocornea, anterior segment dysgenesis, iridocorneal dysgenesis, glaucoma, cataract)	N, S	[146]
**45**	*TMX3*	616102	Microphthalmia with coloboma 1 (MCOPCB1)	300345	A, M	I, C (coloboma, cyst)	N, S	[147]
**46**	*YAP1*	606608	Coloboma, ocular, with or without hearing impairment, cleft lip/palate, and/or mental retardation (COB1)	120433	A, M	I, C (coloboma, extraocular muscle defect, cataract, ectopic pupil)	N, S	[148]
**47**	*IPO13*	610411	-	-	M	C (coloboma, cataract, narrowed palpebral fissure, nystagmus, microcornea)	N	[149]
**48**	*PITX3*	602669	Cataract 11, multiple types (CTRCT11)	610623	M	C (cataract/corneal opacity)	S	[150]
**49**	*NDP*	300658	Norrie disease (ND)	310600	M	C (sclerocornea)	N	[92]
**50**	*MITF*	156845	COMMAD syndrome	617306	M	C (coloboma, microcornea with pannus, cataract, translucent irides, optic nerve/tract hypoplasia)	S	[151]
**51**	*FOXC1*	601090	-	-	M	C (microcornea, sclerocornea, cyst, myopia, cataract, Rieger anomaly, retinal detachment)	N	[152]
**52**	*CRPPA*	614631	Muscular dystrophy-dystroglycanopathy (congenital with brain and eye anomalies), type A, 7 (MDDGA7)	614643	M	C (cataract, optic nerve hypoplasia)	S	[153]
**53**	*FANCL*	608111	Fanconi anemia, complementation group L (FANCL)	614083	M	C (short upslant palpebral fissure, indiscernible pupil)	S	[154]
**54**	*SMO*	601500	Curry-Jones syndrome (CRJS)	601707	M	C (coloboma, unusually shaped pupil)	S	[155]
**55**	*DOCK6*	614194	Adams-Oliver syndrome 2 (AOS2)	614219	M	I, C (retinal detachment)	S	[156]
**56**	*CRYAA*	123580	Cataract 9, multiple types (CTRCT9)	604219	M	C (congenital cataract)	N	[157]
**57**	*FOXL2*	605597	Blepharophimosis, ptosis and epicanthus inversus (BPES)	110100	M	C (blepharophimosis, ptosis, epicanthus inversus, telecanthus, strabismus)	N, S	[158]
**58**	*CRYBA4*	123631	Cataract 23, multiple types (CTRCT23)	610425	M	C (cataract, enophthalmia)	N	[159]
**59**	*ERCC6*	609413	Cerebrooculofacioskeletal syndrome 1 (COFS1)	214150	M	C (congenital cataract, short palpebral fissure, blepharokeratoconjunctivitis)	S	[160,161]
**60**	*ERCC5*	133530	Cerebrooculofacioskeletal syndrome 3 (COFS3)	616570	M	C (cataract)	S	[162]
**61**	*ERCC1*	126380	Cerebrooculofacioskeletal syndrome 4 (COFS4)	610758	M	C (blepharophimosis)	S	[163]
**62**	*SRD5A3*	611715	Congenital disorder of glycosylation, type 1q (CDG1q)	612379	M	C (coloboma, nystagmus, cataract, optic atrophy)	S	[164]
**63**	*SALL4*	607343	Duane-radial ray syndrome (DRRS)	607323	M	C (coloboma, optic nerve hypoplasia)	S	[165]
**64**	*FREM2*	608945	Fraser syndrome 2 (FRASRS2)	617666	M	C (coloboma, cyst)	S	[166]
**65**	*RPGRIP1L*	610937	Meckel syndrome 5 (MKS5)	611561	M	I	S	[167]
**66**	*SLC25A24*	608744	Fontaine progeroid syndrome (FPS)	612289	M	I	S	[168]
**67**	*FAM111A*	615292	Gracile bone dysplasia (GCLEB)	602361	M	I	S	[169]
**68**	*SMG9*	613176	Heart and brain malformation syndrome (HBMS)	616920	M	I	S	[170]
**69**	*SIX3*	603714	Holoprosencephaly 2 (HPE2)	157170	M	C (coloboma, myopia, astigmatism, dysplastic optic nerve, nystagmus, exotropia, cataract, hypertropia)	S	[171]
**70**	*PDE6D*	602676	Joubert syndrome 22 (JBTS22)	615665	M	I, C (coloboma)	S	[172]
**71**	*KMT2D*	602113	Kabuki syndrome 1 (KABUK1)	147920	M	C (cyst)	S	[173]
**72**	*PAX2*	167409	Papillorenal syndrome (PAPRS)	120330	M	C (coloboma, optic nerve dysplasia, retinal degeneration)	S	[174,175]
**73**	*TMEM216*	613277	Meckel syndrome, type 2 (MKS2)	603194	M	I	S	[176]
**74**	*CEP290*	610142	Meckel syndrome, type 4 (MKS4)	611134	M	I	S	[177]
**75**	*KIF11*	148760	Microcephaly with or without chorioretinopathy, lymphedema, or mental retardation (MCLMR)	152950	M	I, C (coloboma, cataract, chorioretinopathy, hypermetropia, persistent hyaloid artery, peripheral fibrovascular proliferation, retinal detachment)	S	[178,179]
**76**	*SNX3*	605930	-	-	M	I	S	[180]
**77**	*ZEB2*	605802	Mowat-Wilson syndrome (MOWS)	235730	M	C (cataract, retinal aplasia, corectopia, optic nerve hypoplasia/pallor, retinal atrophy)	S	[181]
**78**	*POMT1*	607423	Muscular dystrophy-dystroglycanopathy (congenital with brain and eye anomalies), type A, 1 (MDDGA1)	236670	M	C (anterior chamber dysgenesis, exophthalmia, buphthalmos, megalocornea, glaucoma, retinal dysplasia, congenital cataract/corneal clouding, retinal detachment)	S	[182,183]
**79**	*POMT2*	607439	Muscular dystrophy-dystroglycanopathy (congenital with brain and eye anomalies), type A, 2 (MDDGA2)	613150	M	C (Peter’s anomaly, cataract, buphthalmos)	S	[184]
**80**	*POMGNT1*	614828	-	-	M	C (corneal clouding/cataract)	S	[185]
**81**	*FKTN*	607440	Muscular dystrophy-dystroglycanopathy (congenital with brain and eye anomalies), type A, 4 (MDDGA4)	253800	M	C (retinal detachment)	S	[186]
**82**	*FKRP*	606596	Muscular dystrophy-dystroglycanopathy (congenital with brain and eye anomalies), type A, 5 (MDDGA5)	613153	M	C (cataract, asymmetric pupils, persistent hyperplastic primary vitreous, anterior chamber abnormality)	S	[187,188]
**83**	*DAG1*	128239	Muscular dystrophy-dystroglycanopathy (congenital with brain and eye anomalies), type A, 9 (MDDGA9)	616538	M	C (buphthalmos, corneal opacity, glaucoma, retinal detachment)	S	[189,190]
**84**	*B3GALNT2*	610194	Muscular dystrophy-dystroglycanopathy (congenital with brain and eye anomalies, type A, 11 (MDDGA11)	615181	M	C (cataract, optic nerve hypoplasia, myopia)	S	[191]
**85**	*RAB3GAP1*	602536	Warburg micro syndrome 1 (WARBM1)	600118	M	C (microcornea, cataract)	S	[192]
**86**	*RAB3GAP2*	609275	Warburg micro syndrome 2 (WARBM2)	614225	M	C (congenital cataract, small pupil, aphakia, hypermetropia, secondary glaucoma)	S	[193,194]
			Martsolf syndrome	212720				
**87**	*NHS*	300457	Nance-Horan syndrome (NHS)	300672	M	C (microcornea, congenital cataract)	S	[195]
			Cataract 40, X-linked (CTRCT40)	302200				
**88**	*HMX1*	142992	Oculoauricular syndrome (OCACS)	612109	M	C (microcornea, coloboma, nystagmus, cataract, microphakia, synechiae, anterior segment dysgenesis, small dysplastic optic disc, strabismus, sclerocornea, posterior embryotoxon, stromal iris cyst, retinal dystrophy, dysplastic macropapillae, macular hypoplasia, irido-corneal adherences, enophthalmus, esotropia, calcified phthisis bulbi)	S	[196,197]
**89**	*GJA1*	121014	Oculodentodigital dysplasia, autosomal recessive	257850	M	I, C (cataract, uveitis, glaucoma, persistent pupillary membrane)	S	[198,199,200]
			Oculodentodigital dysplasia (ODDD)	164200				
**90**	*LRP5*	603506	Osteoporosis-pseudoglioma syndrome (OPPG)	259770	M	C (retinal detachment, persistent hyperplasia of the primary vitreous)	S	[201]
**91**	*PQBP1*	300463	Renpenning syndrome (RENS1)	309500	M	C (coloboma)	S	[202]
**92**	*TUBB*	191130	Symmetric circumferential skin creases, congenital, 1 (CSCSC1)	156610	M	C (short palpebral fissure, retinal dysplasia, microcornea)	S	[203,204]
			Cortical dysplasia, complex, with other brain malformations 6 (CDCBM6)	615771	M			
**93**	*MAPRE2*	605789	Symmetric circumferential skin creases, congenital, 2 (CSCSC2)	616734	M	C (short/slanting palpebral fissure, strabismus, ptosis)	S	[203]
**94**	*SALL1*	602218	Townes-Brocks syndrome 1 (TBS1)	107480	A, M	C (abnormal lens, aplastic optic nerve, small optic chiasm)	S	[205]
**95**	*HDAC6*	300272	Chondrodysplasia with platyspondyly, distinctive brachydactyly, hydrocephaly, and microphthalmia	300863	M	I	S	[206,207]
**96**	*ALX1*	601527	Frontonasal dysplasia 3 (FND3)	613456	M	C (coloboma)	S	[208]
**97**	*RERE*	605226	Neurodevelopmental disorder with or without anomalies of the brain, eye, or heart (NEDBEH)	616975	M	C (coloboma, optic nerve hypoplasia, anisometropia)	S	[209]
**98**	*RAB18*	602207	Warburg micro syndrome 3	614222	M	C (microcornea, congenital cataract, small atonic pupil, progressive optic atrophy)	S	[210]
